# Midwives: Essential guardians in the climate crisis journey

**DOI:** 10.18332/ejm/188196

**Published:** 2024-05-22

**Authors:** Deepti Ganapathy, Maria Tzeli, Victoria Vivilaki

**Affiliations:** 1 Centre for Management Communication, Indian Institute of Management, Bangalore, India; 2 Midwifery Department, School of Health and Care Sciences, University of West Attica, Athens, Greece; 3 Department of Midwifery, School of Health and Care Sciences, University of West Attica, Athens, Greece

**Keywords:** midwifery, climate crisis, advocacy, sustainability, environmental health, community education

This year, the theme of the International Day of the Midwife 2024 is ‘Midwives: A Vital Climate Solution’.

At the 28th Conference of Parties (COP) summit in Dubai, held in December 2023, a notable development occurred: a dedicated day to health within the COP framework. The health pavilion at COP28 garnered significant attention, positioning itself as a direct consequence of climate change.

Over the years, political leaders, businesses, health organizations, and advocacy groups have convened at COP meetings to push for consensus on Climate Action. By spotlighting a day dedicated to health, COP28 provided a crucial platform for highlighting the health implications of climate change. Communicating this message effectively to relevant stakeholder groups has been a key challenge in the ongoing discussions surrounding climate change and its impact on health.

With the proclamation ‘Midwives: A Vital Climate Solution’, the International Confederation of Midwives (ICM), representing 108 member associations in 98 countries, underscores a sobering reality: a global shortage of 900000 midwives persists, compounded by discrimination, not enabling working conditions, and unequal pay within the profession^1^.

In the midst of a climate crisis, characterized by uncertainty and geopolitical unrest, vulnerable populations, including climate refugees and those below the poverty line, often bear the brunt of its effects. In such situations, midwives emerge as indispensable first responders, rushing aid to women and children in extreme climate events or conflict zones, thereby fulfilling Sustainable Development Goals (SDGs) 2 and 5.

Despite their critical role, midwives are frequently side-lined from health services leadership, policy-making, and stewardship. Creating an enabling environment for midwives is imperative, necessitating strategic litigation for improved salaries. The PUSH movement, spanning a decade, advocates for the rights and autonomy of women and midwives alike^2-5^.

The work of midwives forms the bedrock of women’s reproductive rights^6-8^. Yet, millions of lives are lost annually in childbirth due to the undervaluation and under prioritization of midwives’ skills. From providing access to contraception for 220 million women and girls to averting the needless deaths of 2.7 million women and newborns annually during pregnancy and childbirth, midwives save lives and champion women’s health activism^1^.

As we grapple with the challenge of meeting basic health needs and integrating every healthcare provider into a dignified healthcare infrastructure, the current climate crisis and global warming have cast a glaring spotlight on our healthcare systems^9^. The impact of climate change on human health presents a multifaceted challenge that will require sustained efforts for years to come^10^.

Midwives can contribute to addressing climate change-related health challenges through advocacy, education, and other interventions within healthcare systems^11-15^:

1. Advocacy and Education: Midwives can advocate for policies and initiatives aimed at reducing greenhouse gas emissions by educating pregnant women and new mothers about environmentally friendly practices and encouraging them to adopt sustainable lifestyles^16-20^.
2. Renewable Energy and Clean Transportation: Midwives can promote the use of renewable energy sources and advocate for clean transportation options within healthcare facilities. They can also educate women about the benefits of using clean energy and transportation methods for their own health and the environment^21-23^.
3. Climate-Resilient Infrastructure: Midwives can participate in planning and implementing climate-resilient infrastructure within healthcare settings to ensure that facilities are prepared for extreme weather events. They can also educate women about the importance of accessing healthcare facilities that are resilient to climate change impacts^24-28^.
4. Water and Food Security: Midwives can advocate for improved water and food security measures to reduce malnutrition and related health problems among pregnant women and children. They can provide education on nutrition and promote sustainable farming practices within communities^29-30^.
5. Strengthening Healthcare Systems: Midwives play a crucial role in strengthening healthcare systems to better respond to climate-related health risks, including infectious diseases and mental health issues. They can advocate for increased resources and support for healthcare facilities and provide training on climate-related health risks to healthcare providers^31^.
6. Promoting Sustainable Lifestyles: Midwives can promote sustainable lifestyles by educating women about the health impacts of the climate crisis and fostering behavioral changes to reduce carbon footprints. They can provide information on eco-friendly products and practices that promote health and well-being^32-35^.

The media have a critical role to play in communicating this for policy making and influencing key stakeholders. Studies have shown how newspaper coverage on this creates awareness and behavioral change for key influencers^36^.

Overall, midwives can contribute to addressing the adverse effects of the climate crisis on human health by advocating for policies and initiatives, educating women and communities, and actively participating in efforts to build resilience and promote sustainability within healthcare systems.

## Data Availability

Data sharing is not applicable to this article as no new data were created.
